# Glycocalyx as Possible Limiting Factor in COVID-19

**DOI:** 10.3389/fimmu.2021.607306

**Published:** 2021-02-22

**Authors:** Patricia P. Wadowski, Bernd Jilma, Christoph W. Kopp, Sebastian Ertl, Thomas Gremmel, Renate Koppensteiner

**Affiliations:** ^1^Division of Angiology, Department of Internal Medicine II, Medical University of Vienna, Vienna, Austria; ^2^Department of Clinical Pharmacology, Medical University of Vienna, Vienna, Austria; ^3^Department of Internal Medicine I, Landesklinikum Mistelbach-Gänserndorf, Mistelbach, Austria

**Keywords:** glycocalyx, heparan sulfate, complement, SARS-CoV-2, COVID-19

The coronavirus disease 2019 (COVID-19) has emerged as a global burden comprising cardiovascular and respiratory complications. Herein, endothelial cell infection causing endotheliitis is discussed as a key mechanism. However, knowledge on underlying molecular pathways is still scarce. In this opinion we would like to highlight the role of glycocalyx disturbance for endothelial cell infection possibly being the limiting factor to SARS-CoV-2 disease exacerbation.

Besides the development of an acute respiratory distress syndrome (ARDS) in patients who are critically ill after infection with SARS-CoV-2, endothelial dysfunction seems to be an underlying cause of multiorgan failure. In fact, SARS- CoV-2 directly infects human vascular organoids *in vitro* (1) and autopsy studies of patients dying from coronavirus disease 2019 (COVID-19) show severe endothelial cell damage with disrupted cell membranes, intracellular virus and endotheliitis (2, 3).

The glycocalyx is a key regulator of endothelial cell homeostasis, tissue oedema and inflammatory processes (4). It consists of membrane-bound proteoglycans and glycoproteins and covers endothelial cells at the luminal vessel side (4, 5). Together with adsorbed molecules from the blood plasma, the glycocalyx forms the endothelial surface layer (4). This fragile barrier is disturbed in inflammatory processes (6) and cardiovascular diseases (7–9) being associated with patient outcome (10–12). Recently, it was shown that glycocalyx thickness is predictive of mortality in septic patients, with higher perfused boundary regions (PBR) measured within 24 h after ICU admission in non-survivors (12). Perfused boundary regions were visualized using sublingual non-invasive sidestream-darkfield imaging and used as an indirect and inverse marker of the glycocalyx (12).

In addition, during septic shock, plasma levels of glycosaminoglycans (GAGs) increase suggesting glycocalyx destruction (6, 13) with higher plasma levels of hyaluronan and heparan sulfate (HS) in non-survivors (6). Similarly, urinary levels of hyaluronic acid and HS are higher in non-surviving patients (10). Furthermore, the levels of urinary GAGs predicted the development and progression of renal dysfunction in patients with septic shock and were also associated with in-hospital mortality in patients with ARDS (10). Finally, another component of the glycocalyx, syndecan-1, is elevated continuously in septic non-survivors, while it decreases during the course of the disease in surviving patients (14).

The current SARS-CoV-2 induced COVID-19 is associated with similar patterns of disease exacerbation, namely sepsis, renal failure, and ARDS (3, 15). Since the glycocalyx has a main role in the development of tissue oedema according to the Starling equation, it has been postulated that glycocalyx disintegrity impacts the development of ARDS (16).

Intriguingly, beside binding to angiotensin-converting enzyme 2 (ACE2) (17), SARS-CoV needs HS, the major component of GAGs in the glycocalyx (18), as adhesion molecule (19). The suggested binding site of HS is constituted of positively charged amino acid residues at the receptor-binding domain adjacent to the ACE-2 binding site (20).

SARS-CoV has a similarity to human coronavirus NL63 (HCoV-NL63), an alphacoronavirus, which also uses ACE2 and HS for cellular entry (21). In fact, HS proteoglycans act as adhesion molecules thereby increasing HCoV-NL63 density on the cellular surface (21). The use of glycans as attachment (co-) factors is also observed in the initial interaction of numerous viruses with host cells (22).

The influenza virus, adenovirus, rotavirus, and reovirus bind to sialic acid via stalk-like attachment proteins (23). Herpes simplex virus type 1 (HSV-1) uses galectin-3 as entry mediator (24). In addition, HSV-1 cellular entry depends on HS and especially 3-O-sulfation of specific HS glucosamine residues (25). For HSV-2 the HS entry site is virtually inactive (26). Both HSV-1 and HSV-2 use nectin-1, which interacts with the glycosaminoglycans of the glycocalyx, as entry receptor (26).

Human immunodeficiency virus 1 (HIV-1) binds to HS in a complex manner, where HS can promote viral attachment and transcytosis through epithelia (27). A specific CD4-HS glycoconjugate has been developed to inhibit HIV-1 attachment (27).

In addition, enteroviruses use HS for attachment to host cells (28–30). HS binding is also used by different hepatitis viruses (31–33), human papillomavirus (34), polyomaviruses (35, 36), rhinoviruses (36–38) and coxsackie viruses (39, 40).

Finally, also arthropod-borne viruses like the chikungunya, yellow fever, Japanese and Murray Valley encephalitis virus, eastern equine encephalitis virus, West Nil and Dengue virus are dependent on the glycocalyx as entry site (41–48).

This finding is surprising, as it questions the protective role of the glycocalyx. However, for HCoV-NL63 these observations were seen *in vitro* among others by adding HS proteoglycans to the cell culture (21). *In vivo*, glycocalyx degradation and release of soluble HS is part of the innate immunity, as it is recognized as danger- associated molecular pattern (DAMP), hereby promoting an inflammatory burst by toll-like receptor interaction (49). Hence glycocalyx destruction seems to mediate severe COVID-19. Recently, Stahl et al. demonstrated that COVID-19 patients experience an acquired loss of the protective heparanase 2 and an increase in PBR (50). However, future research should be conducted to give further insights into the natural course of disease pathology and the role of HS. In addition, current studies should emphasize on mutations in the receptor-binding domain of SARS-CoV-2 as for e.g., the N501Y mutation (51) and potential effects on HS binding.

The glycocalyx is a very fragile structure, regulated by shear forces and strongly susceptible to environmental changes (4). Therefore, it has for years been underestimated, because applied staining protocols degraded its structure (4). Furthermore, the endothelial layer is determined by the interaction of the glycocalyx with different plasma proteins (4). In cell cultures, glycocalyx formation is modified by cell type, cellular density, culture conditions and shear stress (52). The latter influences glycocalyx structure and composition (52). Viral cell entry of SARS pseudovirus was reported to be inhibited by lactoferrin, which binds to HS (19).

Hence we assume that glycocalyx disintegrity accounts for enhanced viral entry. It is known, that inflammatory conditions lead to severe glycocalyx modulations, including the shedding of its components like HS, chondroitin sulfate or syndecan and glypican core proteins with attached GAGs (52). Recent reports suggest enhanced heparanase activity in more severe COVID-19 disease (53).

Further, glycocalyx shedding can be conferred by cytokines and chemoattractants (52, 54). The latter, i.e., the complement system was discussed as key factor in SARS- CoV-2 mediated ARDS (55).

Activation of the complement cascade develops 1 day after SARS-CoV infection (56). Herein, activation of C3 has a key role in disease exacerbation, as C3 deficient mice experienced less respiratory dysfunction and weight loss (56).

Following extensive modifications by D-glycuronyl C5-epimerase and 2-O-, 3-O-, and 6-O-sulfotransferases HS is characterized by a heterogenous structure (57). HS structural variability accounts for e.g., for either activation or inhibition of the complement system (57). The grade of sulphation determines C3b cleaving, which is accelerated by HS with lower sulphation (58).

Potential therapeutic approaches include the administration of the glycoprotein SPARC, which has been reported to restore the glycocalyx in coxsackievirus-B3 induced myocarditis (59). Further, albumin infusions have been shown to preserve the glycocalyx and mediate its recovery (60, 61). Albumin carries sphingosine-1 phosphate to the endothelium, which inhibits syndecan-1 shedding (62). Further, rhamnan sulfate, a polysaccharide extracted from the green algae M. nitidum has antiviral and antithrombotic properties (63, 64). Sevoflurane, which is used during anesthesia, has been shown to be protective against ischemia- reperfusion injury of the glycocalyx (65). Recently, liposomal nanocarriers with pre-assembled glycocalyx have been developed and shown to restore NO- production in heparanase III -treated endothelial cells (64, 66). Another therapeutic opportunity includes an anti-adhesive coating of the glycocalyx, consisting of a dermatan sulfate backbone with multiple selectin- binding peptides, which prevents platelet binding to inflamed endothelium (67). This coating reduced *in vivo* thrombus formation in a mouse model of deep vein thrombosis (67).

Heparin, which is closely related to HS has further been speculated to be protective, while binding SARS-CoV-2 and inducing its conformational change (68). Further, heparin acts as heparanase inhibitor (11). However, the grade of sulphation might limit its beneficial effects regarding C3b cleavage and complement activation (58). In addition, heparin interferes with the binding of antithrombin III to the glycocalyx (69). Antithrombin III is known to reduce inflammation (69–71) and to protect the glycocalyx from enzymatic degradation (69).

Another heparanase inhibitor, sulodexide, which is a glycosaminoglycan extracted from porcine intestinal mucosa, was already applied in patients with type 2 diabetes and increased retinal and sublingual glycocalyx thickness (11, 72).

In pre-clinical models the application of atrasentan, a selective endothelin A receptor antagonist, increased glycocalyx dimensions, NO concentrations and reduced heparanase expression and albuminuria (73).

As the inflammatory response is more and more regarded as crucial in COVID-19 exacerbation, the use of steroids, might be helpful to preserve glycocalyx structure in severe cases (74–76). The application of dexamethasone is already recommended in patients who require supplemental oxygen or mechanical ventilation (77). However, adverse effects on glycocalyx structure and endothelial permeability of steroid application have also been reported (78, 79).

In summary, though to date knowledge on SARS-CoV-2 pathogenesis is still scarce, histological findings show endotheliitis and recent *in vivo* measurements suggest endothelial dysfunction as integral element in severe COVID-19 (2, 3, 50).

In consequence we would like to urge researchers to focus on preservation models for glycocalyx composition to enhance benefits for patient outcome.

## Author Contributions

All authors listed have made a substantial, direct and intellectual contribution to the work, and approved it for publication.

## Conflict of Interest

The authors declare that the research was conducted in the absence of any commercial or financial relationships that could be construed as a potential conflict of interest.
